# Endocannabinoid and AGE Interactions in Prediabetes: The Role of Mediterranean Diet Adherence

**DOI:** 10.3390/nu17152517

**Published:** 2025-07-31

**Authors:** Marko Grahovac, Marko Kumric, Marino Vilovic, Daniela Supe-Domic, Nikola Pavlovic, Josipa Bukic, Tina Ticinovic Kurir, Josko Bozic

**Affiliations:** 1 Department of Endocrinology, Diabetes and Metabolic Diseases, University Hospital of Split, Spinciceva 1, 21000 Split, Croatia; marko.grahovac@mefst.hr (M.G.); tina.ticinovic.kurir@mefst.hr (T.T.K.); 2 Department of Pathophysiology, University of Split School of Medicine, Soltanska 2A, 21000 Split, Croatia; marko.kumric@mefst.hr (M.K.); marino.vilovic@mefst.hr (M.V.); nikola.pavlovic@mefst.hr (N.P.); 3 Laboratory for Cardiometabolic Research, University of Split School of Medicine, Soltanska 2A, 21000 Split, Croatia; 4 Department of Medical Laboratory Diagnostics, University Hospital of Split, Spinciceva 1, 21000 Split, Croatia; daniela.supe.domic@ozs.unist.hr; 5 Faculty of Health Sciences, University of Split, Rudera Boskovica 35, 21000 Split, Croatia; 6 Department of Pharmacy, University of Split School of Medicine, Soltanska 2A, 21000 Split, Croatia; josipa.bukic@mefst.hr

**Keywords:** anandamide, prediabetes, advanced glycation end-products, mediterranean diet

## Abstract

**Objectives**: To determine whether plasma concentrations of anandamide (AEA) and 2-arachidonoylglycerol (2-AG) are elevated in adults with prediabetes, we explored their association with tissue advanced glycation end-products (AGEs) and assessed the influence of Mediterranean diet adherence. **Methods**: This cross-sectional single-centre study included 92 adults with prediabetes and 86 age-/sex-matched normoglycaemic controls. Anthropometry, blood pressure, biochemical indices, and skin autofluorescence-derived AGEs were measured. Serum AEA and 2-AG were quantified by competitive ELISA, while Mediterranean diet adherence was assessed using the Mediterranean Diet Serving Score (MDSS). **Results**: Prediabetes was associated with higher AEA (*p* = 0.004) but not 2-AG (*p* = 0.520). Also, AEA correlated positively with AGE values (*r* = 0.36; *p* = 0.002) and increased across AGE-based cardiovascular risk categories. In multivariable models, both prediabetes status and AGE burden independently predicted AEA. Participants achieving MDSS ≥ 14 exhibited lower AEA (*p* = 0.038); 2-AG remained unaffected. Finally, the multivariable analysis confirmed that both prediabetes (β = 11.9; *p* = 0.005) and AGE values (β = 0.25; *p* = 0.003) are positively associated with plasma AEA levels, independent of age, sex, BMI, and fasting plasma glucose levels. **Conclusions**: Circulating AEA, but not 2-AG, is elevated in prediabetes and independently linked to cumulative AGE burden, suggesting early endocannabinoid activation contributes to cardiometabolic risk. High adherence to a Mediterranean diet may mitigate this dysregulation.

## 1. Introduction

Prediabetes represents the initial identifiable stage of glucose dysregulation, serving as a transitional phase from normoglycemia to type 2 diabetes. This condition is characterized by impaired fasting glucose (IFG) and/or impaired glucose tolerance (IGT), indicating not only an elevated risk for the development of type 2 diabetes but also an increased likelihood of associated complications [1]. As of 2021, the age-adjusted global prevalence of IGT and IFG was estimated at 9.1% (464 million individuals) and 5.8% (298 million individuals), respectively, among adults aged 20 to 79 years. Projections suggest that within the next two decades, these figures could rise to 10.0% (638 million) for IGT and 6.5% (414 million) for IFG. Consequently, as the global incidence of prediabetes escalates, it becomes imperative to elucidate the underlying mechanisms that contribute to insulin resistance and metabolic dysregulation [2,3].

One of the essential pathological processes in a patient with prediabetes is the accelerated formation of advanced glycation end products (AGEs), which arise from the non-enzymatic glycation of proteins, lipids, and nucleic acids due to hyperglycemia [4]. AGEs are implicated in the pathophysiology of insulin resistance, inflammation, endothelial dysfunction, and oxidative stress, thereby exacerbating risk factors associated with various metabolic disorders [5]. Furthermore, endocannabinoids, principally anandamide (AEA) and 2-arachidonoylglycerol (2-AG), have emerged as dynamic lipid messengers that couple nutritional status, immune signalling, and nociception [6]. Synthesized on demand from membrane phospholipid precursors, they act locally and are rapidly inactivated by fatty acid amide hydrolase (FAAH) or monoacyl-glycerol lipase (MAGL), thereby providing finely tuned, short-lived signals. These endogenous compounds interact with the endocannabinoid system (ECS), which consists of three primary components: G-protein coupled cannabinoid receptors (CB1 and CB2), their respective ligands, and the enzymes responsible for their synthesis and degradation [6,7]. Through CB1 activation, AEA and 2-AG stimulate lipogenesis, reduce adiponectin release, modulate insulin secretion, and influence hepatic gluconeogenesis, thereby linking caloric excess to weight gain and insulin resistance. Conversely, peripheral CB2 engagement dampens cytokine production and leukocyte recruitment, limiting low-grade inflammation that accompanies metabolic dysregulation [7,8]. Biochemical and structural studies consistently describe AEA and 2-AG as the best-studied endocannabinoid ligands, accounting for the majority of cannabinoid signalling observed in brain and peripheral tissues [6,7,8].

Recent research indicates that energy imbalances associated with obesity and hyperglycemia lead to heightened expression of cannabinoid receptors alongside enzymes involved in their synthesis and degradation. This cascade initiates a series of pathophysiological changes consistent with the metabolic disturbances linked to elevated levels of AGEs [9,10]. Moreover, studies have shown that blockade of the CB1 receptor yields beneficial outcomes in animal models of obesity and metabolic syndrome, findings that have been explored in human populations as well [11,12]. Elevated concentrations of AEA and 2-AG have been correlated with increased inflammation and insulin resistance, while AGEs have been demonstrated to activate inflammatory pathways that may also influence the ECS [13,14,15]. Despite these mechanistic overlaps, most studies have investigated the ECS, AGEs, and diet-derived modulators in isolation, leaving a critical gap in our understanding of how nutritional patterns might simultaneously influence both axes. The Mediterranean diet (MD), characterized by high intakes of extra-virgin olive oil, nuts, legumes, fruits, vegetables, and moderate wine consumption, has emerged as a leading dietary approach for cardiometabolic prevention, owing in part to its polyphenol-rich, anti-inflammatory, and antioxidant profile [16,17]. Still, only a few investigations have examined whether adherence to a Mediterranean diet can modulate circulating endocannabinoid levels or mitigate AGE accumulation, particularly in populations at the earliest stages of dysglycaemia.

Key components of the Mediterranean diet, such as polyphenols and monounsaturated fatty acids, may mechanistically influence both the ECS and AGE pathways. Polyphenols, abundant in extra-virgin olive oil, fruits, and vegetables, exhibit antioxidant and anti-inflammatory effects that reduce oxidative stress, a major driver of AGE formation. Polyphenols also modulate the ECS by inhibiting enzymes like FAAH and MAGL, altering endocannabinoid degradation and receptor signalling, which may improve insulin sensitivity and reduce inflammation [18]. Monounsaturated fats, especially oleic acid from olive oil, influence membrane lipid composition and the biosynthesis of endocannabinoids such as 2-AG and anandamide, potentially normalizing ECS tone under metabolic stress. Together, these diet-derived compounds may synergistically attenuate ECS overactivation and limit AGE accumulation, offering metabolic protection in at-risk prediabetic populations [19,20].

The principal aim of the present study was therefore to determine whether plasma concentrations of 2-AG and AEA are associated with AGE levels in adults with prediabetes, and to explore whether varying degrees of Mediterranean diet adherence are linked to parallel changes in both biomarkers. We hope that, by addressing this intersection, we can provide new insight into the combined role of diet, ECS activity, and AGE burden in this patient population.

## 2. Materials and Methods

### 2.1. Study Design and Ethical Considerations

This research was designed as a cross-sectional single-centre study performed at the Regional Center for Diabetes, Endocrinology, and Metabolic Diseases of the University Hospital of Split (Split, Croatia) from December 2024 to February 2025. The study was conducted in accordance with the latest adaptation of the Helsinki Declaration and was approved by the Ethics Committee of the University of Split School of Medicine (Class: 029-01/24-02/0001; No: 2181-198-03-04-24-0111; Date: 2 December 2024). The researchers thoroughly briefed the participants on the procedures and purpose of the study, and each participant personally signed an informed consent form.

### 2.2. Participants

A total of 178 participants were included, 92 patients with a diagnosis of prediabetes, and 86 healthy age- and sex-matched control participants. The sample size was determined a priori to ensure adequate statistical power for detecting differences between groups. This calculation was based on data obtained from a pilot investigation, which included 10 participants per group. Using these pilot data, the required sample size was established to achieve a statistical power of 80% with an alpha error rate of 5%. The analysis indicated that 70 participants per group are necessary to provide sufficient statistical validity for the main study.

The inclusion criterion was a diagnosis of prediabetes according to the American Diabetes Association’s latest criteria: fasting plasma glucose level between 5.6 and 6.9 mmol/L or hemoglobin A1c level between 5.7% and 6.4% or oral 2 h plasma glucose level during an oral glucose tolerance test between 7.8 and 11.0 mmol/L [21]. Exclusion criteria were: diagnosis of diabetes (of either type), significant chronic heart, kidney, or liver disease, previous myocardial infarction or ischemic stroke, established peripheral artery disease, secondary forms of arterial hypertension, active malignant disease, acute infection, autoimmune disease, taking a supplementation that contains cannabis in any form (marijuana smoking included), current corticosteroid therapy and any other therapy or disease that could significantly disrupt glucose plasma level (e.g., Cushing syndrome, acromegaly, pheochromocytoma). Clinical information for each participant was gathered through anamnesis and a review of their medical records during the screening visit. Control participants were screened using the same exclusion criteria.

### 2.3. Anthropometric Measurements

Body weight and height were recorded with a calibrated scale-stadiometer (Seca, Birmingham, UK). BMI was calculated as weight/height^2^. Waist and hip circumferences were taken with a 0.5 cm-precision tape at the midpoint between the iliac crest and the costal margin and at the widest gluteal point, respectively; the waist-to-hip ratio was then calculated.

### 2.4. Arterial Blood Pressure and Advanced Glycation End Products (AGEs)

Office blood pressure was measured with an OMRON M3 Comfort (Omron Healthcare Co., Ltd., Kyoto, Japan) following current ESC recommendations [22]. Skin autofluorescence, a proxy for accumulated advanced glycation end products (AGEs), was assessed non-invasively on the right forearm with the AGE Reader (DiagnOptics Technologies BV, Groningen, The Netherlands). This method closely matches dermal AGE content in biopsies [23]. As the signal is affected by melanin, only participants with Fitzpatrick I–III skin types were enrolled. Three readings were averaged, and the device’s software classified cardiovascular risk as “no/limited” or “increased/definite” (combined for analysis). The technique’s intra-individual coefficient of variation is <5%.

### 2.5. Laboratory Evaluations

Venous samples provided a full blood count, lipid panel, liver enzymes, fasting glucose, HbA1c, urea, creatinine, and electrolytes. Plasma levels of the endocannabinoids anandamide (AEA) and 2-arachidonoylglycerol (2-AG) were quantified using a competitive enzyme-linked immunosorbent assay (ELISA). Following thawing, plasma samples were kept on ice to minimize enzymatic degradation. Prior to analysis, samples were prediluted at a ratio of 1:4 for AEA and 1:5 for 2-AG using phosphate-buffered saline (PBS; pH 7.2), in accordance with the manufacturer’s instructions. Quantification was performed using commercial ELISA kits (Abbexa, Cambridge, UK), and absorbance was measured on an Elisys Duo ELISA analyser (Human Diagnostics Worldwide). The intra-assay and inter-assay coefficients of variation were <10% for AEA and <12% for 2-AG, indicating good assay precision and reproducibility. Laboratory technicians performing the ELISA and data analysts involved in statistical evaluations were blinded to group assignments throughout the study to minimize bias.

### 2.6. IPAQ-SF and MDSS Survey

Participants completed two validated questionnaires: the 14-item Mediterranean Diet Serving Score (MDSS) and the short-form International Physical Activity Questionnaire (IPAQ-SF) [24,25]. The MDSS is a validated 14-item questionnaire that evaluates adherence to the Mediterranean diet in accordance with the latest guidelines of the Mediterranean Diet Pyramid. Participants were instructed to report their typical dietary habits over the past year to reflect long-term adherence. This tool assesses the frequency of consumption of specific foods and food groups, assigning 3 points to foods eaten at every meal (fruits, vegetables, cereals, and olive oil), 2 points to daily items (dairy and nuts) and 1 point to weekly items (white/red meat, fish, potatoes, legumes, eggs, sweets, and wine). Finally, ≥14 points signifies Mediterranean diet adherence. The IPAQ-SF is a validated open-ended questionnaire that records walking, moderate, and vigorous activity over the previous 7 days. Energy expenditure is expressed as MET-minutes/week using 3.3, 4.0, and 8.0 METs, respectively, summed for a total score.

### 2.7. Statistical Analysis

All statistical analyses were performed using SPSS Statistics version 30.0 (IBM Corp., Armonk, NY, USA) and GraphPad Prism version 10.0 (GraphPad Software, San Diego, CA, USA). A two-sided *p*-value < 0.05 was considered statistically significant. The normality of data distribution was assessed using the Shapiro–Wilk test. Continuous variables are presented as mean ± standard deviation (SD) for normally distributed data and as median with interquartile range (IQR) for non-normally distributed data. Categorical variables are shown as counts with percentages. Comparisons between two independent groups were performed using Student’s *t*-test for normally distributed continuous variables, the Mann–Whitney U test for non-normally distributed continuous variables, and the chi-square (χ^2^) test for categorical variables. Associations between endocannabinoid levels and continuous clinical or biochemical parameters were assessed using Spearman’s rank correlation analysis. Finally, to determine whether prediabetes and AGE levels are independently associated with plasma AEA levels, a multiple linear regression analysis was performed. The multivariable model adjusted for potential confounders, including age, sex, body mass index (BMI), and fasting plasma glucose. Variables that demonstrated significance in univariable analyses were considered for inclusion in the multivariable model.

**Figure 1:**
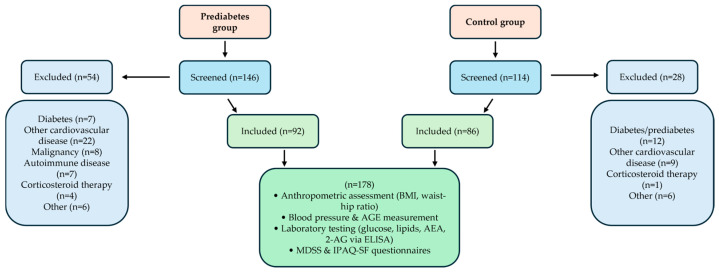
Flowchart of participant screening, inclusion, and assessment procedures for prediabetes and control groups.

Figure 1 illustrates the selection process for study participants divided into two groups: the Prediabetes group and the Control group. Initially, 146 individuals were screened for the Prediabetes group, out of which 54 were excluded due to criteria including diabetes, other cardiovascular diseases, malignancy, autoimmune diseases, corticosteroid therapy, or other reasons, resulting in 92 participants included in the study. For the Control group, 114 individuals were screened, with 28 excluded for reasons such as diabetes/prediabetes, cardiovascular diseases, corticosteroid therapy, or other causes, leading to 86 participants included.

Both groups combined account for a total of 178 participants. All included subjects underwent anthropometric assessments (body mass index and waist-hip ratio), blood pressure and advanced glycation end products (AGE) measurements, laboratory testing (glucose, lipids, AEA, and 2-AG via ELISA), and completed MDSS and IPAQ-SF questionnaires.

## 3. Results

Table 1 presents a comparison of baseline characteristics between patients diagnosed with prediabetes and healthy age- and sex-matched controls. Patients with prediabetes had higher fasting plasma glucose (*p* = 0.024), HbA1c (*p* = 0.031), BMI (*p* = 0.032), and systolic and diastolic blood pressure (*p* < 0.001 and *p* < 0.001, respectively) compared to normoglycemic counterparts. Additionally, a higher proportion of patients with prediabetes were classified as having increased AGE-based cardiovascular risk compared to controls (41 (44.6%) vs. 23 (26.7%), *p* = 0.013). Other baseline characteristics did not differ between the groups of interest.

**Figure 2:**
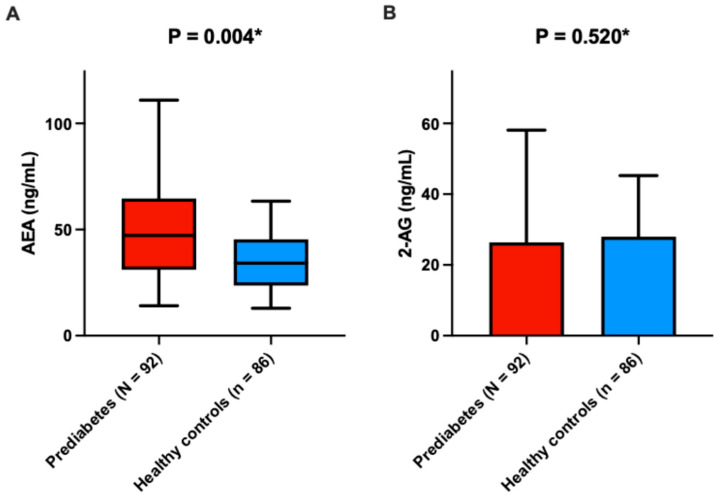
Comparison of endocannabinoid plasma levels between patients with prediabetes and normoglycemic controls. (**A**) AEA; (**B**) 2-AG. Abbreviations: AEA: anandamide; 2-AG: 2-arachidonoylglycerol. * Mann–Whitney U test.

Patients with prediabetes exhibited higher plasma levels of AEA in comparison to normoglycemic age- and sex-matched controls (47.2 (31.1–64.0) ng/mL vs. 34.2 (23.6–44.8) ng/mL, *p* = 0.004, rank-biserial *r* = −0.34, 95% CI [−0.50, −0.16]) (Figure 2).

No such difference between patients with prediabetes and normoglycemic controls was found for 2-AG plasma levels (25.5 ± 9.2 ng/mL vs. 26.9 ± 11.4 ng/mL, *p* = 0.520, *d* = 0.06, 95% CI [−0.28, 0.40]) (Figure 2B).

**Figure 3:**
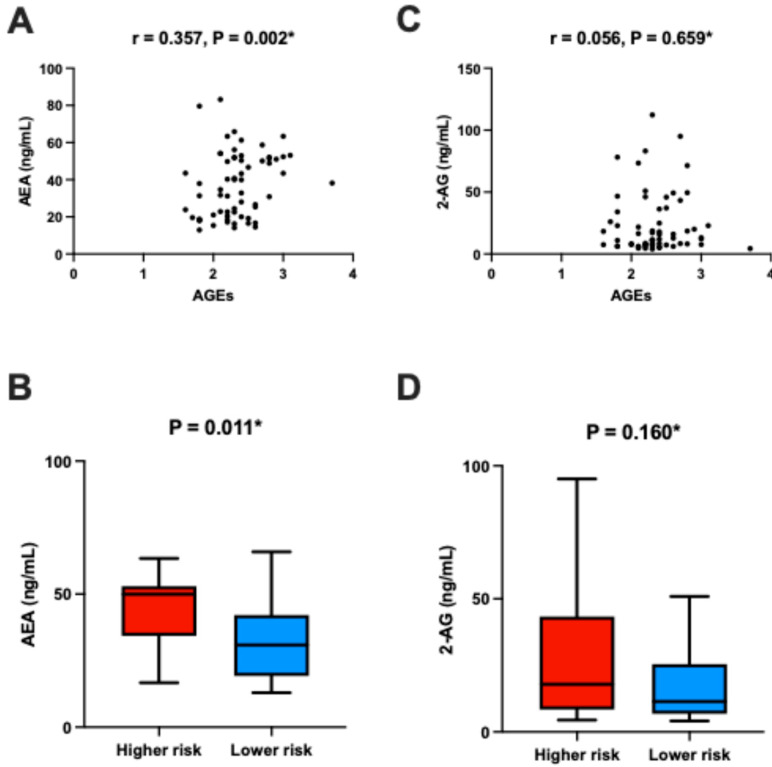
Association between plasma AEA levels and AGEs: (**A**) Correlation between plasma AEA and AGE values. (**B**) Plasma AEA levels stratified by AGE-based cardiovascular risk categories. (**C**) Comparison of plasma AEA levels between the higher-risk and lower-risk groups. (**D**) Comparison of plasma 2-AG levels between the higher-risk and lower-risk groups. The lower-risk group includes participants classified as having “No cardiovascular risk” or “Limited cardiovascular risk,” while the higher-risk group includes those with “Increased cardiovascular risk” or “Definite cardiovascular risk,” based on the AGEReader manufacturer’s original stratification. * Spearman’s rank correlation analysis.

Univariable analysis revealed a positive correlation between plasma AEA levels and AGE values (*r* = 0.357, *p* = 0.002) (Figure 3A). This finding is further supported by the observation that patients with higher AGEs-based cardiovascular risk exhibited elevated plasma AEA levels compared to those in the lower-risk group (50.0 (38.2–53.0) ng/mL vs. 30.9 (19.4–41.5) ng/mL, *p* = 0.011, rank-biserial *r* = −0.39, 95% CI [−0.62, −0.11]) (Figure 3B). No such associations were found for 2-AG plasma levels (11.4 (6.9–25.2) ng/mL vs. 17.9 (8.5–41.8) ng/mL, *p* = 0.158, rank-biserial *r* = −0.21, 95% CI [−0.47, 0.08]) (Figure 3D). Furthermore, no correlation was found between plasma 2-AG levels and AGE values (*r* = 0.056, *p* = 0.659) (Figure 3C).

Patients adherent to the Mediterranean diet had lower AEA levels compared to their non-adherent counterparts (*p* = 0.038), whereas no such association was found for 2-AG levels. In contrast, no association was identified between adherence to individual food groups of the Mediterranean diet and plasma levels of either AEA or 2-AG (Table 2).

Correlation analysis between plasma AEA, 2-AG, and other relevant clinical and biochemical parameters is presented in Appendix A Table A1. The univariable analysis showed that age positively correlates with AEA (*p* < 0.001), but not 2-AG levels (*p* = 0.180).

Finally, in order to determine whether prediabetes and AGEs are independently associated with plasma AEA levels, a multiple linear regression analysis was employed. The multivariable analysis confirmed that both prediabetes and AGE values are positively associated with plasma AEA levels, independent of age, sex, BMI, and fasting plasma glucose levels (Table 3).

## 4. Discussion

In the present study, we demonstrated that patients with prediabetes exhibit higher plasma levels of AEA compared to normoglycemic age- and sex-matched controls. This result is consistent with earlier studies indicating that the endocannabinoid system (ECS) may be dysregulated in metabolic diseases. The review by Nogueiras et al. highlights the role of the ECS in glucose and energy metabolism, noting that AEA can influence insulin secretion and sensitivity [26]. The ECS, located at both central and peripheral levels, regulates the communication between the brain and peripheral organs, adjusting the activity of tissues involved in lipid and glucose metabolism [27]. Activation of CB1 cannabinoid receptors in peripheral tissues such as the liver, adipose tissue, and skeletal muscle can lead to metabolic dysregulation, including impaired insulin signalling, increased hepatic gluconeogenesis, and increased lipogenesis [28]. Elevated AEA levels in prediabetes may reflect compensatory mechanisms in response to insulin resistance [26]. In addition, this finding also aligns with previous research indicating that elevated levels of AEA have been correlated with coronary circulatory dysfunction in human obesity [29], a condition closely linked to prediabetes through mechanisms involving excess visceral adiposity, insulin resistance, impaired glucose metabolism, and many others [30]. Similarly, increased AEA levels have been found in the retina of patients with diabetic retinopathy, suggesting AEA as a potential biomarker linking metabolic dysfunction with vascular complications of diabetes [31]. Moreover, the four multicentric and multinational Rimonabant In Obesity (RIO) trials showed that treatment with a CB1 antagonist in humans resulted in a reduction in body weight and waist circumference, along with improvements in several cardiometabolic risk factors such as triglyceride levels, fasting insulin, glycated hemoglobin (HbA1c), and blood pressure, accompanied by favourable increases in HDL cholesterol and adiponectin concentrations [32,33,34,35]. However, the drug mentioned was withdrawn from the market after regulatory agencies, including the FDA and EMA, raised concerns about its safety, particularly due to psychiatric side effects [26]. Interestingly, 2-arachidonoylglycerol (2-AG) levels were not different between prediabetic individuals and controls. This is consistent with findings from other studies, which suggest that AEA and 2-AG are differentially regulated and may play distinct roles in cardiometabolic diseases [36,37]. These divergent findings likely stem from fundamental differences in biosynthesis and degradation pathways; AEA is primarily hydrolyzed by fatty acid amide hydrolase (FAAH), whereas 2-AG is predominantly metabolized by monoacyl-glycerol lipase (MAGL). Variations in tissue-specific enzyme expression, substrate availability, and local metabolic demands may result in distinct circulating levels. Moreover, AEA and 2-AG differ in their affinities and efficacies at CB1 and CB2 receptors, which could translate into differing physiological and pathophysiological roles. This underscores the need to consider each endocannabinoid separately when interpreting their contributions to metabolic regulation.

Higher AEA and heightened ECS activity seem to promote insulin resistance. Annuzzi et al. observed elevated AEA in subcutaneous fat of obese type 2 diabetics, paralleling rises in leptin mRNA and free fatty acids [38]. In mice, Liu et al. showed AEA-driven hepatic CB1 activation causes glucose intolerance, an effect lost in CB1-knockouts [39]. An eight-week human RCT found switching from a Western to an isocaloric Mediterranean diet lowered fasting AEA and improved HOMA-IR, implicating the diet’s monounsaturated fat and polyphenols in dampening ECS overactivity [40]. Complementing this, mechanistic work in 2024 revealed 2-AG-induced CB1 signalling in monocytes upregulates resistin, sparking adipose inflammation and systemic insulin resistance, reversed by CB1 blockade [41].

Together, these findings suggest that dietary modulation of the ECS may attenuate downstream pro-inflammatory mediators such as resistin, yet the extent to which this axis operates in individuals with prediabetes, and whether high adherence to a Mediterranean diet can simultaneously lower AEA/2-AG, resistin, and AGE burden, remains to be further elaborated.

Plasma AEA levels positively correlated with advanced glycation end products (AGEs) tissue values, a previously recognized marker of cumulative metabolic stress and microvascular damage [42]. Accordingly, multivariable analysis confirmed that prediabetes and AGE values are independently associated with plasma AEA levels, even after adjusting for potential confounding factors such as age, sex, BMI, and fasting plasma glucose levels. Moreover, patients with higher AGEs-based cardiovascular risk had elevated plasma AEA levels compared to those with lower risk. Rajesh et al. suggest that excessive activation of the ECS and CB1 receptor may play an important role in the pathogenesis of diabetic cardiomyopathy, among other things, by facilitating the expression/accumulation/signalling of RAGEs and AGEs [12]. Elevated levels of various AGEs have been identified in the sera and tissues of individuals with diabetes. Numerous studies have previously demonstrated a link between increased production of AGEs and diabetes complications, indicating their role as a potential biomarker and even as predictors of such outcomes [4,5]. While the predictive value of the level of AGEs for cardiovascular risk and diabetes-related complications is well-established in individuals diagnosed with type 2 diabetes, there remains a lack of evidence supporting their prognostic utility in the context of prediabetes. Several studies have explored the association between AGEs and prediabetes, highlighting their potential role as biomarkers and predictors of metabolic dysfunction. A systematic review and meta-analysis conducted by Lu et al. indicated that high levels of exogenous AGEs (dietary intake of AGEs mostly via processing food at high temperatures and low moisture, including grilling, roasting, and frying) is linked to increased fasting plasma glucose, insulin levels, and insulin resistance (HOMA-IR), all of which are key indicators of prediabetes and increased risk for type 2 diabetes [43]. Moreover, Birukov et al. conducted a study that suggests that an increased level of AGEs, measured through skin autofluorescence which is previously shown equivalent to skin biopsy [23], could play a role in vascular stiffening, regardless of age and other cardiometabolic risk factors, affecting not only individuals with diabetes but also in normoglycemic and prediabetic conditions [44]. This suggests that elevated AGE levels may contribute to early vascular changes in prediabetic states. On the other hand, the SALIA study examined the relationship between plasma AGEs and impaired fasting glucose. The study found no differences in the AGE level between individuals with normal fasting glucose and those with impaired fasting glucose, suggesting that AGEs may not be strongly associated with early-stage glucose metabolism dysregulation [45]. Collectively, these findings support the growing body of evidence suggesting that dysregulation of the ECS, particularly AEA signalling, may play a role in early metabolic dysfunction. Previous studies have shown that AEA can modulate insulin sensitivity, inflammation, and endothelial function, all of which are relevant to the pathophysiology of prediabetes, obesity, and atherosclerosis [46,47]. The lack of association between 2-AG and either prediabetes or AGE levels may reflect the distinct metabolic pathways and receptor affinities of AEA and 2-AG [48] or differences in their tissue-specific synthesis and degradation [49].

Adherence to the MD, in general, was associated with lower AEA levels, whereas no such associations were found for 2-AG levels or adherence to individual food groups. This pattern implies that the cumulative effect of the Mediterranean diet’s complex nutrient matrix, rather than isolated food groups, is critical to modulating ECS tone. Synergistic interactions among polyphenols, monounsaturated fats, fibre, and omega-3 fatty acids likely underpin this modulation. Hence, total dietary patterns may exert more substantial biological effects on ECS regulation and AGE burden than single dietary components, emphasizing the translational importance of holistic dietary interventions. This aligns with findings from a randomized controlled trial conducted by Rossi et al., which showed that 8 weeks of MD significantly decreased circulating AEA concentrations, independently of weight loss [50]. The MD’s high content of monounsaturated fatty acids, fibre, and polyphenols lowers endocannabinoid tone [16,51] and confers recognized cardioprotection [16]. These nutrients may act through altered fatty acid profiles, reduced oxidative stress, or shifts in the gut microbiota [40,52]. The MD’s link with AEA, but not 2-AG, parallels animal data showing different fatty-acid control of these mediators [20]. Moreover, omega-3 polyunsaturated fatty acids abundant in the MD further dampen ECS activity and inflammation, strengthening its vascular benefits [53]. Taken together, our results highlight the potential of AEA as a biomarker linking metabolic dysfunction with vascular risk in the population with prediabetes. The observed relationship between AEA and AGEs suggests a role for endocannabinoid signalling in the early stages of cardiometabolic disease, potentially offering new avenues for risk stratification or therapeutic intervention. Importantly, these findings position AEA as a promising early biomarker of metabolic stress and vascular risk in prediabetes. Its sensitivity to pathophysiological changes potentially preceding overt diabetes suggests that monitoring plasma AEA could enhance early detection and risk assessment, guiding tailored preventive strategies. Further longitudinal studies are essential to validate its predictive utility in clinical settings.

### Strengths and Limitations

This study has several strengths. It is among the first to examine the relationship between circulating endocannabinoid levels and advanced glycation end products (AGEs) in a prediabetic population. The inclusion of a well-defined, age- and sex-matched control group, rigorous exclusion criteria, and the use of validated tools such as the MDSS and IPAQ-SF enhance the internal validity of the findings. Furthermore, the blinding of laboratory personnel and data analysts to group assignments minimized the risk of measurement bias.

The present study has limitations that should be acknowledged. While the sample size provides sufficient statistical power for the primary analyses, it may limit the generalizability of the findings to broader populations. Also, the study included only Caucasian participants, which restricts its applicability to other ethnic or racial groups that may exhibit different endocannabinoid profiles or metabolic characteristics. In addition, the single-centre design introduces potential bias related to specific institutional practices or regional variations in lifestyle and dietary habits, which could influence the observed associations. Lastly, the cross-sectional nature of the study precludes the establishment of causal relationships between plasma AEA levels, prediabetes, and AGEs. Thus, longitudinal studies are required to clarify the temporal dynamics and causal pathways underlying these associations. A limitation of the present study is the lack of detailed body composition measurements, such as bioimpedance-derived fat mass or fat percentage. While BMI and waist-to-hip ratio are commonly used and informative indicators of adiposity, emerging evidence highlights the added value of precise body composition assessment in metabolic risk stratification. Future studies incorporating direct evaluation of fat mass and distribution would provide additional insight into the relationships among adiposity, ECS activity, and AGE accumulation in prediabetes.

The voluntary nature of participation may also introduce selection bias, as individuals who choose to participate in research may differ systematically from those who do not. Furthermore, we did not collect detailed information on certain lifestyle variables such as smoking status, alcohol consumption beyond wine, sleep quality, or the use of concomitant medications, which may have influenced the outcomes. Although multivariable models were adjusted for key confounders, the possibility of residual confounding cannot be fully excluded. Lastly, the cross-sectional nature of the study precludes the establishment of causal relationships between plasma AEA levels, prediabetes, and AGEs. Thus, longitudinal studies are required to clarify the temporal dynamics and causal pathways underlying these associations.

## 5. Conclusions

In conclusion, our findings demonstrate that elevated plasma AEA levels are independently associated with prediabetes and markers of cardiovascular risk, primarily the level of AGEs. These results support the hypothesis that the ECS plays an early role in the pathogenesis of metabolic and vascular disease. The observed relationship between AEA and AGEs suggests a role for endocannabinoid signalling in the early stages of cardiometabolic disease, potentially offering new possibilities for risk stratification or therapeutic interventions. Nonetheless, further research is needed to elucidate the mechanisms underlying these associations and to explore the potential of targeting the ECS in the prevention and management of cardiometabolic diseases. On the other hand, dietary modulation of AEA levels via the MD offers a promising, non-pharmacological strategy for reducing cardiovascular risk in this population. To confirm the causality and long-term relevance of these findings, longitudinal and interventional studies are warranted to examine whether adherence to the Mediterranean diet can beneficially modulate ECS and AGE profiles in prediabetic individuals.

## Figures and Tables

**Table 1:** Baseline characteristics of the studied population.

Parameter	Prediabetes (*n* = 92)	Control Group (*n* = 86)	*p*-Value
Age (years)	54.2 ± 8.3	56.6 ± 9.1	0.067 *
Male sex, *n* (%)	38 (41.3)	39 (45.3)	0.694 **
BMI (kg/m^2^)	30.4 ± 3.8	26.4 ± 2.9	0.032 *
WHR	0.99 ± 0.31	0.96 ± 0.32	0.684 *
Systolic BP (mmHg)	134.8 ± 11.9	123.2 ± 9.4	<0.001 *
Diastolic BP (mmHg)	84.9 ± 10.2	78.3 ± 7.6	<0.001 *
FPG (mmol/L)	5.8 ± 0.6	5.1 ± 0.7	0.024 *
HbA1c (%)	5.9 ± 0.4	5.5 ± 0.3	0.031 *
Total cholesterol (mmol/L)	5.7 ± 0.9	5.2 ± 1.1	0.038 *
LDL-C (mmol/L)	3.5 ± 0.7	3.3 ± 1.1	0.142 *
HDL-C (mmol/L)	1.6 ± 0.5	1.4 ± 0.4	0.234 *
Triglycerides (mmol/L)	1.5 ± 0.9	1.2 ± 0.3	0.022 *
AST (U/L)	25.5 ± 7.8	24.1 ± 8.4	0.494 *
ALT (U/L)	26.2 ± 9.3	22.3 ± 9.1	0.382 *
Creatinine (mmol/L)	78 ± 15	75 ± 15	0.765 *
AGE value	2.4 ± 0.5	2.2 ± 0.4	0.104 *

Abbreviations: AGE: advanced glycation end product; ALT: alanine aminotransferase; AST: aspartate aminotransferase; BMI: body mass index; BP: blood pressure; FPG: fasting plasma glucose; HbA1c: glycated hemoglobin; HDL-C: high-density lipoprotein cholesterol; LDL-C: low-density lipoprotein cholesterol; WHR: waist-to-hip ratio. * Student’s *t*-test. ** Chi-squared test.

**Table 2:** Comparison of endocannabinoid plasma values based on adherence to individual food groups and Mediterranean diet in the total study population.

Parameter	AEA (ng/mL)			2-AG (ng/mL)		
	Adherent	Non-Adherent	*p* *	Adherent	Non-Adherent	*p*-Value
Cereals	35.0 (18.3–61.1)	35.7 (21.1–55.1)	0.423	31.6 (23.0–41.1)	30.8 (24.1–39.9)	0.723
Potato	37.5 (19.0–65.1)	38.5 (22.0–61.1)	0.627	19.0 (13.7–29.7)	15.3 (12.5–23.0)	0.063
Olive oil	40.2 (20.2–68.2)	41.0 (23.2–62.3)	0.765	31.0 (22.0–40.6)	29.9 (23.5–39.0)	0.114
Nuts	42.3 (22.3–70.3)	42.9 (25.3–65.0)	0.274	19.2 (10.0–29.0)	20.3 (12.5–28.4)	0.456
Fruit	38.3 (20.3–63.0)	37.8 (24.3–60.1)	0.857	27.6 (18.1–36.6)	26.8 (19.7–36.0)	0.534
Vegetables	41.1 (18.1–65.0)	41.4 (21.0–58.1)	0.539	32.5 (25.7–38.6)	31.9 (26.5–37.9)	0.798
Dairy products	36.5 (15.1–58.2)	38.5 (19.0–54.2)	0.092	21.4 (12.0–30.9)	22.5 (13.1–31.5)	0.555
Legumes	39.2 (17.5–64.1)	38.4 (22.5–59.6)	0.102	27.4 (22.0–33.2)	26.2 (22.1–32.0)	0.364
Eggs	43.6 (23.0–72.6)	43.3 (26.6–66.5)	0.788	19.1 (11.2–29.0)	18.6 (11.9–27.8)	0.277
Fish	37.5 (16.0–61.5)	36.8 (21.7–57.7)	0.234	21.9 (13.6–30.5)	22.4 (15.1–29.9)	0.355
White meat	44.2 (24.2–64.2)	44.0 (25.3–62.3)	0.698	44.4 (37.0–51.3)	43.5 (37.5–50.4)	0.243
Red meat	42.7 (24.3–73.3)	44.9 (27.3–67.0)	0.082	28.7 (22.5–35.8)	29.2 (24.1–36.0)	0.756
Sweets	39.3 (21.3–62.4)	39.8 (24.6–62.1)	0.586	25.7 (12.5–34.0)	26.0 (21.9–34.5)	0.819
Wine	41.5 (18.6–64.0)	43.4 (26.0–57.1)	0.094	32.1 (26.8–40.8)	31.5 (27.4–39.9)	0.745
MD adherence †	38.0 (20.3–51.9)	47.5 (27.7–51.8)	0.038	18.2 (12.8–30.8)	21.5 (16.2–27.9)	0.653

Abbreviations: MD: Mediterranean diet. * Mann–Whitney U test; † According to Mediterranean Diet Serving Score.

**Table 3:** Multivariable analysis of parameters associated with plasma AEA levels.

Variable	β (95% CI) †	SE	*p*-Value
Prediabetes (vs. control)	11.9 (3.7 to 20.1)	4.1	0.005
AGEs (arbitrary units)	0.25 (0.09 to 0.41)	0.08	0.003
Age (years)	0.31 (0.09 to 0.53)	0.11	0.007
Sex (male vs. female)	–1.4 (–8.8 to 6.0)	3.7	0.712
BMI (kg/m^2^)	0.06 (–0.26 to 0.38)	0.16	0.702
Fasting plasma glucose (mmol/L)	–0.88 (–3.9 to 2.1)	1.5	0.561

Abbreviations: AEA: anandamide; AGE: advanced glycation end product; BMI: body mass index; SE: standard error. † multiple linear regression analysis.

## Data Availability

All data and materials are available upon request due to ethical reasons.

## Appendix A

**Table A1:** Correlation analysis between plasma endocannabinoid levels and selected clinical and laboratory characteristics in the total study population (*n* = 178).

Parameters	AEA (ng/mL)		2-AG (ng/mL)	
	*r*—Correlation Coefficient *	*p*	*r*—Correlation Coefficient *	*p*
Age (years)	0.304	<0.001	−0.101	0.180
BMI (kg/m^2^)	0.147	0.050	0.231	0.002
WHR	0.065	0.389	0.124	0.099
Body fat, %	0.101	0.180	0.234	0.002
Visceral fat, %	0.045	0.551	0.121	0.108
Systolic BP (mmHg)	0.065	0.389	0.064	0.396
Diastolic BP (mmHg)	−0.185	0.013	−0.195	0.009
Fasting plasma glucose (mmol/L)	−0.323	<0.001	−0.085	0.259
Total cholesterol (mmol/L)	0.034	0.652	−0.094	0.212
LDL-C (mmol/L)	−0.095	0.207	0.016	0.832
HDL-C (mmol/L)	0.211	0.005	−0.341	<0.001
Triglycerides (mmol/L)	0.114	0.130	0.056	0.458
Creatinine (mmol/L)	0.214	0.004	−0.105	0.163
IPAQ-SF (MET-min/week)	−0.089	0.237	0.312	<0.001
MDSS (points)	−0.203	0.007	0.062	0.437

Abbreviations: * Mann–Whitney U test.
